# Exploring therapeutic applications of *PTEN, TMPRSS2:ERG* fusion, and tumour molecular subtypes in prostate cancer management

**DOI:** 10.3389/fonc.2025.1521204

**Published:** 2025-03-17

**Authors:** Fidelis Charles Bugoye, Rispah Torrorey-Sawe, Richard Biegon, Nazima Dharsee, Fidelice Mafumiko, Herry Kibona, Said Aboud, Kirtika Patel, Simeon Mining

**Affiliations:** ^1^ Directorate of Forensic Science and DNA Services, Government Chemist Laboratory Authority, Dar es Salaam, Tanzania; ^2^ Department of Pathology, Moi University, Moi Teaching and Referral Hospital, Eldoret, Kenya; ^3^ Clinical Research, Training and Consultancy Unit, Ocean Road Cancer Institute, Dar es Salaam, Tanzania; ^4^ Department of Urology, Muhimbili National Hospital, Dar es Salaam, Tanzania; ^5^ Head Office, National Institute for Medical Research, Dar es Salaam, Tanzania

**Keywords:** tumour molecular subtype, *PTEN*, *TMPRSS2:ERG*, genome, immune response, prostate cancer, immunohistochemistry

## Abstract

**Background:**

Prostate cancer is defined by the suppression of genes that suppress tumours and the activation of proto-oncogenes. These are the hallmarks of prostate cancer, and they have been linked to numerous genomic variations, which lead to unfavourable treatment outcomes. Prostate cancer can be categorised into various risk groups of tumour molecular subtypes grounded in the idea of genomic structural variations connected to *TMPRSS2:ERG* fusion and loss of *PTEN*. Research suggests that certain genomic alterations may be more prevalent or exhibit different patterns in prostate cancer tumours across populations. Studies have reported a higher frequency of *PTEN* loss and *TMPRSS2:ERG* fusion in prostate tumours of Black/African American men, which may contribute to the more aggressive nature of the disease in this population. Thus, therapeutically important information can be obtained from these structural variations, including correlations with poor prognosis and disease severity.

**Methods:**

Peer-reviewed articles from 1998 to 2024 were sourced from PubMed and Google Scholar. During the review process, the following search terms were employed: “Tumour suppressor genes OR variations OR alterations OR oncogenes OR diagnostics OR ethnicity OR biomarkers OR prostate cancer genomics OR prostate cancer structural variations OR tumour and molecular subtypes OR therapeutic implications OR immunotherapy OR immunogenetics.”

**Results:**

There was a total of 13,012 results for our search query: 5,903 publications from Google Scholar with the patent and citation unchecked filer options, and 7127 articles from PubMed with the abstract, free full text, and full-text options selected. Unpublished works were not involved. Except for four articles published between 1998 and 1999, all other selected articles published in 2000 and later were considered. However, papers with irrelevant information or redundant or duplicate content were not chosen for this review. Thus, 134 met the inclusion criteria and were ultimately retained for this review.

**Conclusion:**

This review extracted 134 relevant articles about genomic structure variations in prostate cancer. Our findings demonstrate the importance of *PTEN* and *TMPRSS2:ERG* fusion and tumour molecular subtyping in prostate cancer precision medicine.

## 1 Introduction

Prostate cancer (PCa) is one of the most prevalent cancers worldwide and a leading cause of cancer-related deaths among men (1, 2). Its diverse clinical presentation and unpredictable prognosis make it a significant public health concern. Developments in molecular oncology have drawn attention to how important genetic and molecular alterations are in promoting the advancement of PCa and resistance to treatment. These include deletion of the *PTEN* tumour-suppressor, *TMPRSS2:ERG* gene fusion, and other molecular subtypes that have become important biomarkers with significant effects on treatment and prognosis (3–5).

This review explores the therapeutic potential of targeting *ERG/PTEN* molecular subtypes emphasising their role in advancing personalised medicine for PCa treatment. The *TMPRSS2:ERG* fusion, one of the most frequent genetic alterations in PCa, occurs in 40%–50% of cases (3–5). This fusion produces a distinct molecular subtype linked to intermediate-to-aggressive disease symptoms, with the androgen-responsive *TMPRSS2* promoter driving the overexpression of the *ERG* transcription factor (3–5). PCa progression is significantly influenced by *ERG* changes as well as the loss or inactivation of *PTEN*, a key regulator of the PI3K/AKT signalling pathway. *PTEN* deletions are found in approximately 40% of primary PCa cases and are significantly more common in castration-resistant and metastatic forms of the illness (3–5). The PI3K/AKT signalling pathway is uncheckedly activated when *PTEN* is lost, which promotes tumour development, survival, and resistance to treatment. The interplay between *TMPRSS2:ERG* fusion (often represented by *ERG* expression) and *PTEN* loss allows for the classification of PCa into molecular subtypes providing critical insights into tumour biology (4). It is noteworthy that tumours with both *PTEN* loss and *ERG* expression exhibit distinct clinical characteristics and therapeutic responses, indicating the potential of integrating these biomarkers to enhance personalised treatment strategies (6, 7). Understanding PCa heterogeneity has advanced significantly with the development of molecular subtyping based on *ERG* and *PTEN* expression (6, 7). These subtypes not only increase prognosis accuracy but also facilitate tailored therapies, like medicines that target *ERG*-mediated pathways or PI3K/AKT inhibitors for tumours losing *PTEN* (6, 7). Even though the roles of *ERG* and *PTEN* in PCa progression have been the focus of several studies, a thorough synthesis of current studies is required to completely clarify its clinical consequences (4, 6, 8).

This review aims to clarify the roles of *ERG* and *PTEN* molecular subtypes in PCa development and progression emphasising their potential as biomarkers for prognosis, detection, and targeted therapy. By integrating existing evidence, we seek to provide a groundwork for advancing personalised treatment approaches in PCa.

## 2 Materials and methods

### 2.1 Search strategy and data sources

Peer-reviewed articles from 1999 to 2024 were sourced from PubMed and Google Scholar. During the review process, the following search terms were employed: “Tumour suppressor genes OR variations OR alterations OR oncogenes OR diagnostics OR ethnicity OR biomarkers OR prostate cancer genomics OR prostate cancer structural variations OR tumour and molecular subtypes OR therapeutic implications OR immunotherapy OR immunogenetics.”

### 2.2 Selection and search results

There were 13,030 total results for our search query: 5,903 publications from Google Scholar with the patent and citation unchecked filer options and 7,127 articles from PubMed with the abstract, free full text, and full-text options selected.

### 2.3 Inclusion criteria

The filtering method also yielded irrelevant results due to the overly broad key phrases in the search string. The following criteria for inclusion were applied during the manual screening of the obtained articles: 1) Articles discussing the phenotypic or genetic disorders associated with PCa, 2) publications detailing genetic changes or variants linked to PCa, 3) articles outlining changes in tumour-suppressor genes and oncogenes related to PCa, 4) articles exploring how genes associated with PCa may influence prognosis and diagnosis, 5) articles on the clinical applications of tumour subtyping and PCa risk classification, and 6) tumour-suppressor genes and oncogenes in the context of PCa treatments. Unpublished works were excluded. Except for four articles published between 1998 and 1999, all other selected articles published in 2000 and later were included. However, papers containing irrelevant information or redundant or duplicate content were not considered for this review. Thus, 134 articles were ultimately retained for this review.

## 3 Molecular alteration in prostate cancer

The initiation, development, progression, and treatment resistance of PCa are driven by a complex interplay of genetic and molecular alterations reflecting the disease’s inherent heterogeneity (9, 10). Among the most well-reported and clinically relevant molecular alterations in PCa are the loss of *PTEN* and the *TMPRSS2:ERG* gene fusion. The *TMPRSS2:ERG* fusion leads to the overexpression of the *ERG* transcription factor, a key driver of tumour invasion and progression (9, 10). Similarly, *PTEN*, a tumour-suppressor gene that regulates the PI3K/AKT signalling pathway, is commonly lost or inactivated, particularly in advanced and metastatic tumours. The co-occurrence of these alterations is strongly associated with resistance to conventional therapies, aggressive tumour behaviour, and poor clinical outcomes. Advances in understanding the biology and clinical implications of *ERG* and *PTEN* alterations have illuminated the pathophysiology of PCa and paved the way for the development of targeted and personalised therapeutic strategies. These insights are crucial for improving patient outcomes and addressing the challenges posed by the disease’s heterogeneous nature.

### 3.1 Phosphatase and tensin homolog (*PTEN*)

Structurally, *PTEN* is located at the 10q23 locus on chromosome 10, with nine exons and eight introns. It is roughly 200-kb long and codes for a 403-amino acid multifunctional protein that has lipid phosphatase activity (9, 10). *PTEN* dephosphorylates phosphoinositide substrates to create a dual-specific protein phosphatase that is essential for controlling the PI3K/AKT signalling pathway (9, 10). This regulation affects essential cellular functions such as apoptosis, cell cycle regulation, cell invasion inhibition, and general tumour suppression (9, 10). Approximately 50% of castration-resistant prostate tumours frequently exhibit *PTEN* mutations, deletions, and inactivation, which contribute to dysregulated PI3K/AKT signalling (7). As a result, the onset, spread, and poor clinical outcomes of PCa are all strongly associated with the loss of *PTEN* function (9, 10).

#### 3.1.1 The nuclear function of PTEN

PTEN, the most frequently altered tumour-suppressor gene (TSG) in PCa, plays a significant role in maintaining genomic stability through its nuclear activities (11). While PTEN is known for its ability to suppress tumours by preventing the oncogenic PI3K signalling pathway, the available findings suggest that its tumour-suppressive functions extend beyond its lipid phosphatase activity (12). Previous studies hypothesised that PTEN functions as a lipid phosphatase in the nucleus due to the presence of PI3K, AKT, and pyruvate dehydrogenase kinase 1 enzyme (PDK1) in this compartment (13). In addition, subsequent research has demonstrated that PTEN’s nuclear functions are not primarily regulated by its lipid phosphatase activity, as its main substrate, PIP3, is not significantly present in the nucleus (14). Given that several anti-PI3K medications are unable to completely suppress tumour growth in PTEN-deficient malignancies, available data suggest that PTEN has tumour-suppressive effects that extend beyond PI3K pathway inhibition (15, 16). Specifically, nuclear PTEN localises to heterochromatin, contributing to structural stability and reinforcing its phosphatase-independent tumour-suppressive roles (17). These findings highlight the multifaceted nature of PTEN’s functions and underscore its importance in both cytoplasmic and nuclear contexts.

#### 3.1.2 Cytoplasmic functions of PTEN

Due to its dual-specificity phosphatase activity, *PTEN* operates in the cytoplasm to dephosphorylate phosphoinositol (3,4,5)-trisphosphate (PIP3), thereby preventing AKT activation and directly suppressing PI3K signalling (9). The PI3K pathway is typically activated when growth factors bind to receptor tyrosine kinases triggering the conversion of PIP2 to PIP3. This process promotes AKT activation through phosphorylation mediated by PDK1 (18). Once activated, AKT modulates several downstream targets, including mTOR, which enhance cell growth, proliferation, and survival (19).

**Figure 1:**
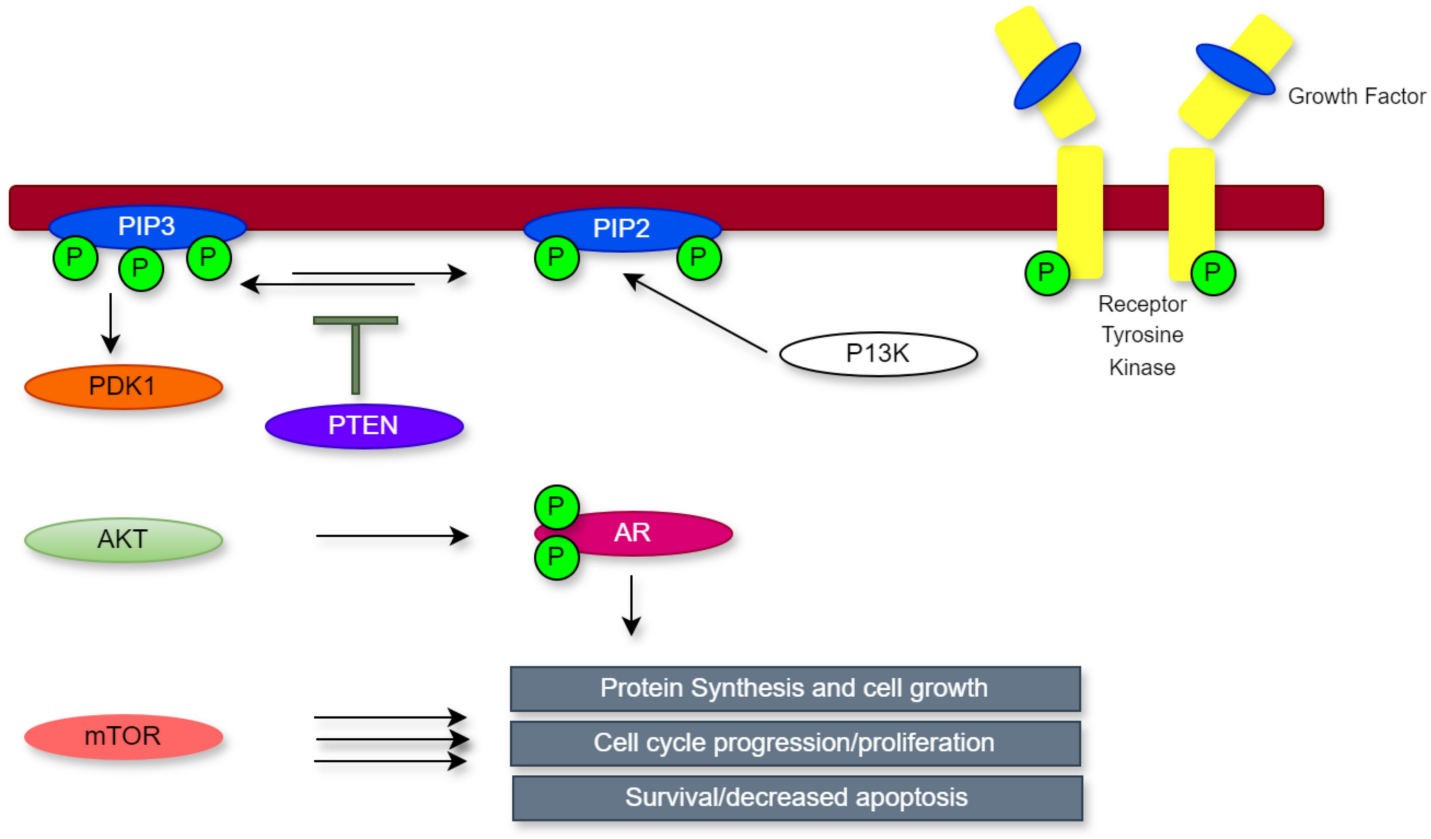
The *PI3K/PTEN/AKT* pathway.

This regulatory mechanism is disrupted when *PTEN* is lost, which results in uncontrolled PI3K/AKT signalling. In PCa, this dysregulation contributes to resistance to androgen deprivation therapy by promoting androgen-independent activation of the androgen receptor (AR) pathway. Furthermore, *PTEN* inactivation leads to a reduced control over vital cellular functions that are essential to the growth of tumours, such as energy metabolism, cell survival, proliferation, and structural integrity (18). In addition, as seen in **Figure 1**, *PTEN* is essential for the development and spread of cancer. These findings underscore the multifaceted contributions of *PTEN* to tumour biology and highlight its significance as a key regulator of cellular homeostasis and cancer progression.

#### 3.1.3 Loss of PTEN gene and immunosuppression in PCa

**Figure 2:**
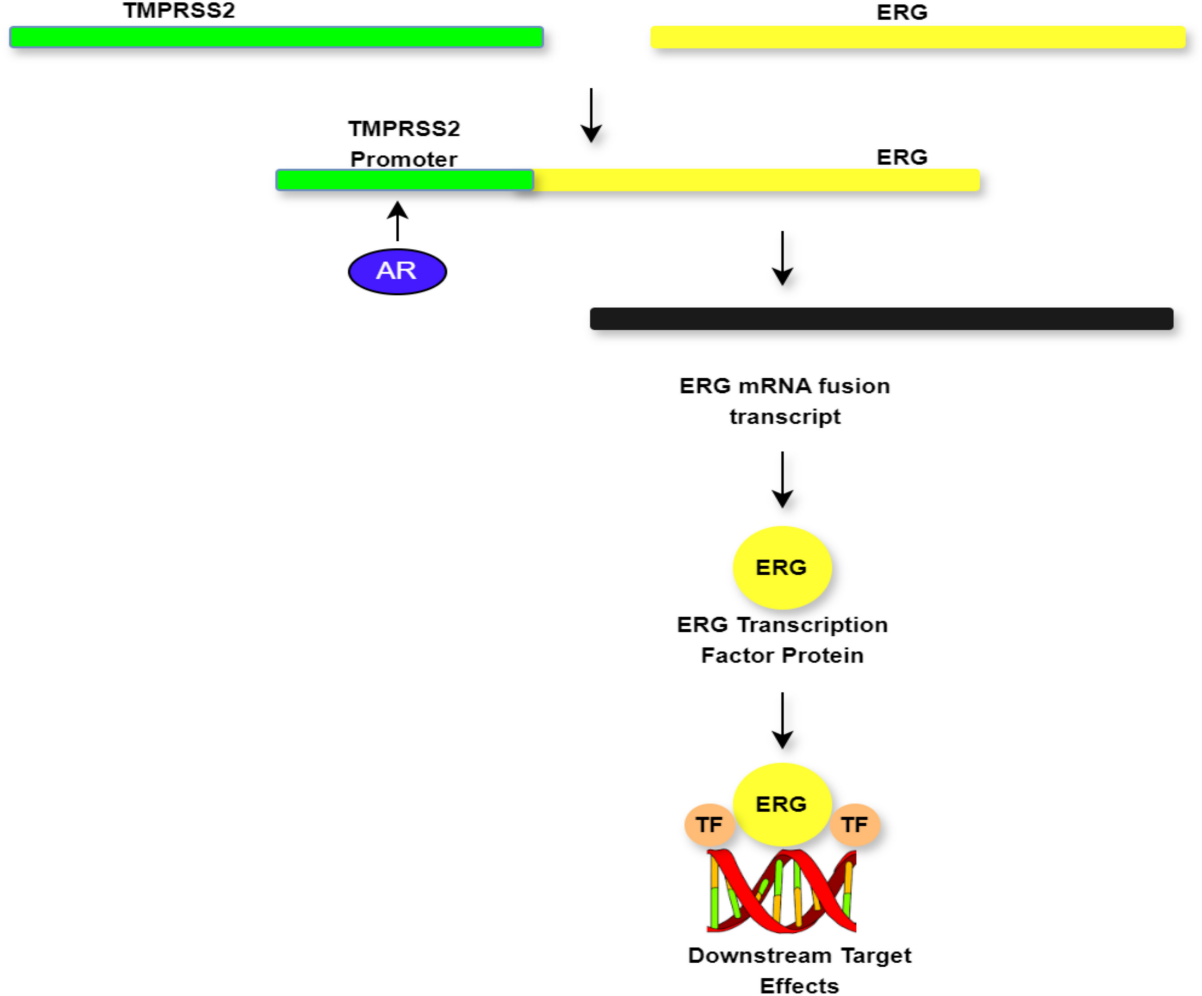
Demonstration of the biology of *TMPRSS2:ERG* fusion and potential actionable drug targets.

The primary mechanism of *PTEN* loss in PCa is copy number variations, which differentiate PCa from other types of malignancies (20). Loss of *PTEN* function drives metabolic reprogramming influencing aggressive tumour growth and rapid cell proliferation (9, 21). This dysfunction affects chromatin structure leading to the loss of heterochromatic foci, reduced chromatin compaction, amplification of heterochromatic genes, disruption of heterochromatin protein 1, and, eventually, genomic instability and loss of *PTEN* function (22). Loss of *PTEN* function activates the PI3K–AKT signalling pathway, which is strongly connected with poor PCa outcomes, as illustrated in **Figure 2**. Available evidence suggests that *PTEN* could function as a genetic marker to differentiate aggressive PCa from indolent PCa, especially in clinically localised PCa (9, 23). Furthermore, *PTEN* facilitates tumour formation by modulating the tumour microenvironment (TME) and immune responses (9). The available research findings have shown that loss of *PTEN* function in PCa correlates with higher Gleason scores and advanced tumour stages. However, inconsistencies in reported findings, which are linked to methodological variations, participant selection, and population variability, underscore the need for further research (9, 24). Ethnic differences in *PTEN* loss have also been reported, with African-American men demonstrating lower rates of *PTEN* loss compared to European-American men (25, 26). Despite these insights, the relationship between racial background, *PTEN* loss, and poor prognosis is not well known and warrants further research (26).

*PTEN* loss is closely linked to the development of an immunosuppressive TME characterised by increased cytokine and chemokine signalling. This environment is enhanced with immunosuppressive cells, such as myeloid-derived suppressor cells (MDSCs), regulatory T cells (Tregs), and M2-polarised macrophages, which prevent antitumour immune responses (27). Additionally, *PTEN*-deficient tumours show increased expression of immune evasion markers, including programmed death-ligand 1 (PD-L1) and indoleamine 2,3-dioxygenase 1 (IDO1), which damage the cytotoxic activity of immune cells and facilitate immune escape (18). The loss of *PTEN*’s nuclear functions further exacerbates inflammation and cytokine signalling, contributing to an immunosuppressive TME (27). This milieu, dominated by M2 macrophages, MDSCs, and regulatory immune cells, suppresses antitumour immunity. Moreover, *PTEN* loss is associated with heightened genomic instability, which can generate neoantigens capable of activating CD8+ T cells and triggering an immune response (18). However, infiltrating tumour-associated macrophages may decrease the immune system’s reaction to counteract neoantigen-driven immunity in cancers with high levels of genomic instability (18).

### 3.2 *TMPRSS2:ERG* gene fusion in PCa

The androgen-regulated gene *TMPRSS2* is found on chromosome band 21q22 and is mainly expressed in the prostate (3). The *ERG* gene, also mapped to 21q22, is located approximately 3 Mb downstream of *TMPRSS2*. The *TMPRSS2:ERG* gene fusion, the most prevalent structural variation in PCa, has been reported in nearly half of all PCa cases (28, 29). This fusion is common in both early and advanced stages of the disease underlining its importance in PCa pathogenesis (5, 30, 31). While it is not frequently reported in normal prostate tissue, *TMPRSS2:ERG* fusions facilitate tumour progression by promoting angiogenesis, inflammation, and epithelial–mesenchymal transition ultimately leading to metastasis, advanced tumour stages, and increased mortality (32–34). Other ETS family members, such as ETV1, ETV4, and ETV5, as well as androgen receptor (AR) targets, like SLC45A3 and NDRG, are also implicated in gene fusions in PCa.

The *TMPRSS2:ERG* fusion occurs when androgen and prostate-specific regulatory regions of TMPRSS2 are juxtaposed with the first exon(s) of *ERG* leading to androgen-driven overexpression of the fusion transcript. In nearly 50%–60% of fusion-positive tumours, this results from an intronic deletion on chromosome 21, which deletes the genomic region between *TMPRSS2* and *ERG*. Alternatively, complicated genomic rearrangements involving chromosome 21q22 and other chromosomes may also give this fusion (35–37). The recurring nature of this fusion is associated with a common deletion spot connecting *ERG* and *TMPRSS2* (38). The deletion site is characterised by two types of breakpoints, with the 3′ end of *ERG* consistently fused to the 5′ end of *TMPRSS2*. This fusion leads to the overexpression of *ERG*, facilitated by the androgen-responsive promoter of *TMPRSS2*, resulting in elevated PCa cell invasion, proliferation, angiogenesis, and tumour aggressiveness (39, 40). Moreover, the *TMPRSS2:ERG* fusion triggers downstream oncogenes, further amplifying its carcinogenic effects (41).

Similarly, androgen receptor activation provides a key role in this process by enhancing *TMPRSS2* promoter function leading to the production of an *ERG* mRNA fusion transcript. This transcript is translated into the *ERG* transcription factor, which consequently activates downstream oncogenic signalling pathways (42, 43). This molecular cascade provides two key therapeutic targets. First, androgen receptor inhibitors (e.g., enzalutamide and abiraterone) suppress *TMPRSS2* activation reducing fusion transcript production and *ERG* overexpression (44). Second, bromodomain inhibitors (e.g., JQ1) disrupt the transcriptional machinery required for *ERG*-mediated gene activation, thereby preventing its oncogenic effects (42, 45). These targeted approaches, which interrupt critical nodes in the *TMPRSS2:ERG* fusion pathway, provide potential opportunities for personalised therapy in PCa (46, 47). The mechanisms regulating ERG overexpression in PCa cells are illustrated in **Figure 2**.

#### 3.2.1 Functional *ERG* overexpression in PCa

The transition from prostatic intraepithelial neoplasia (PIN) to PCa is characterised by higher Gleason scores, metastasis, advanced tumour stages, and reduced survival rates. This progression is facilitated by the persistent overexpression of the *ERG* oncogene (28, 48). In contrast to other ETS family members, such as SAM-pointed domain-containing ETS transcription factor (SPDEF) and ETS2 repressor factor (ERF), which are important for maintaining normal prostate epithelium, ERG is only moderately expressed in normal prostate cells (49), though, when overexpressed in prostate cells, *ERG* drives a range of oncogenic effects. For instance, *ERG* interacts with the PI3K oncogenic signalling pathway leading to tumourigenesis (48). Additionally, *ERG* enhances androgen receptor (AR) binding and enhances AR transcription, especially in PCa patients with loss of *PTEN* function (50). Notably, AR binding patterns are knowingly altered in cells with increased *ERG* expression (50, 51). Furthermore, ERG triggers the Wnt signalling pathway, elevating β-catenin activation and facilitating PCa development and progression (52–54). Despite these oncogenic functions, available findings suggest that *ERG* overexpression alone is insufficient to initiate cancer and is not reliably associated with disease progression (6, 48, 50, 55–57). Ethnic and population-based variations in *ERG* rearrangements and increased expression have also been reported (**Table 1**). In American populations, the overall frequency of *ERG* expression ranges between 50% and 55%, with rates of 28% observed among Caucasian Americans. Among African American populations, *ERG* expression rates vary considerably depending on the studied population. Findings from Sub-Saharan Africa remain inadequate. In Asian populations, *ERG* rearrangements appear in 50%–55% of cases, with rates of 28% in Indian patients and 49% in Chinese PCa patients (34). These differences highlight the impact of genetic and environmental factors on ERG-driven oncogenesis.

**Table 1:** Prevalence of individual *ERG* and *PTEN* expression status across the populations.

SN	ERG+ (%)	ERG- (%)	PTEN+ (%)	PTEN- (%)	Population	Techniques used	References
1	40	60	83	17	UK	FISH	(58)
2	53%	–	–	–	USA	IHC	(59)
3	59.3	–	–	42.9	Jordan	IHC	(60)
4	–	–	–	40%	UK	IHC	(61)
5	–	–	–	39	Brazil	FISH	(62)
6	39.6	–	12.6	–	Brazil	IHC	(63)
7	42.7	–	30.6	–	Middle east	IHC	(64)
8	41.5	–	–	63.6	Canada	FISH	(65)
9	35.5	–	–	–	Switzerland	IHC	(66)
10	48.8	–	–	–	Switzerland	IHC	(66)
11	27	–	–	–	Asia	RT-PCR	(67)
12	25	–	–	–	African Ancestries	RT-PCR	(67)
13	18	–	–	–	Ghana	RT-PCR	(67)
14	49				European Ancestries	RT-PCR	(67)
15	28				African American	RT-PCR	(67)
16	13				Black south Africa	RT-PCR	(67)
17			18.3		USA	FISH	(68)
18			20.2		German	FISH	(69)
19			68		Canada	FISH	(70)
20	88				Brazil	FISH	(71)
21				25	Afro American	IHC	(72)
22			47.5	38.1	North eastern Brazil	IHC	(72)
21	75.4				Uganda	IHC	(73)
22				40	Canada	IHC	(9)
23	13				Black South Africa	RNA sequencing	(67)
24	49				Greece	FISH	(74)

Significantly, *ERG* overexpression may function as an indicator of disease aggressiveness, and its interaction with other regulatory pathways, such as loss of *PTEN*, further highlights its role in PCa progression (75, 76). PCa individuals exhibiting *ERG*-positive high-grade prostatic intraepithelial neoplasia (HGPIN) are at a considerably elevated risk of developing PCa emphasising the clinical relevance of *ERG* as a potential biomarker for disease stratification and risk assessment (77).

### 3.3 *ERG* and the androgen receptor interplay in PCa

**Figure 3:**
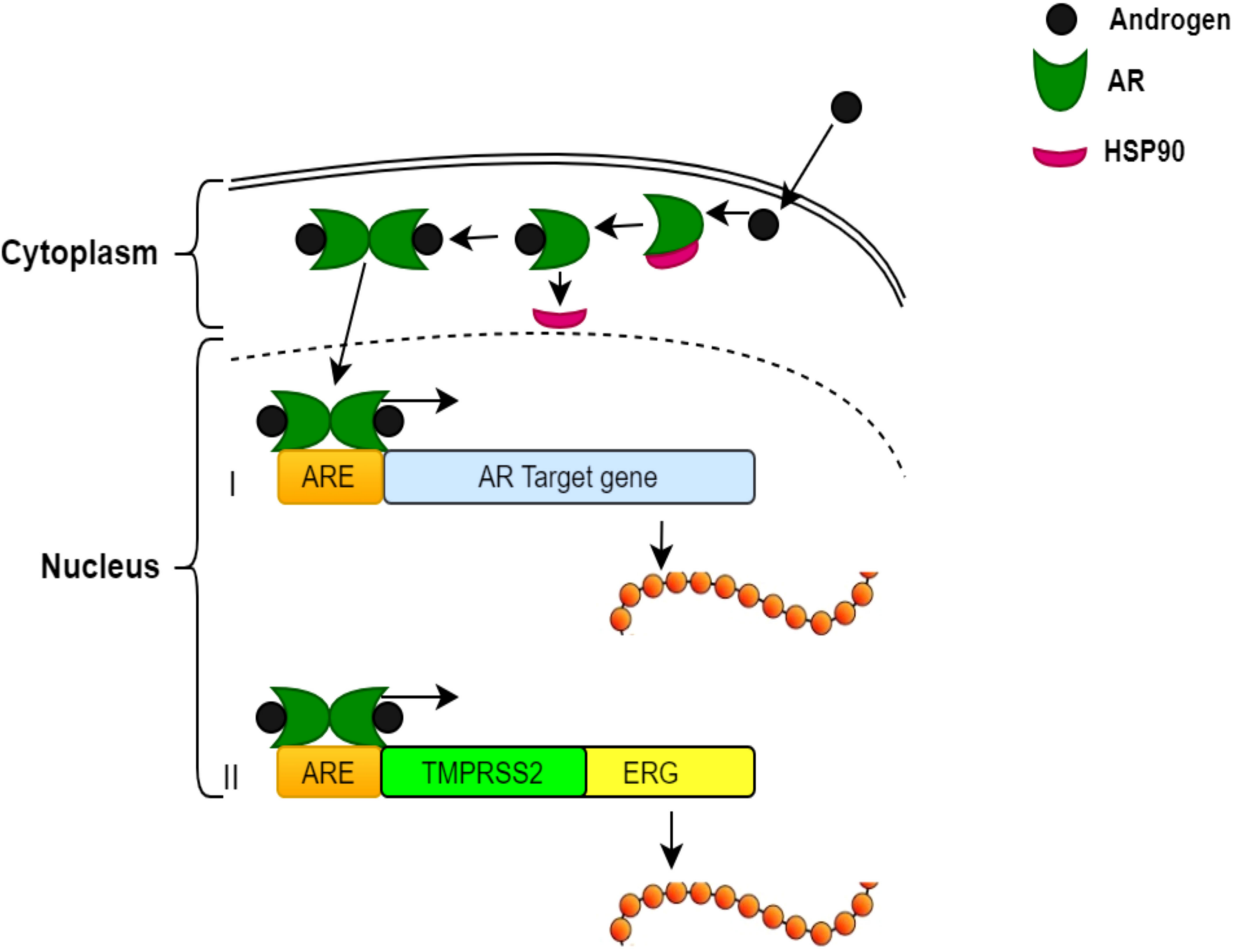
Mechanisms of androgen regulation of gene expression in PCa cells. Inactive AR in the cytoplasm bound to HSP90. The binding of androgens induces a conformational change and activates AR, releasing Hsp90. Activated AR moves to the nucleus. AR binds to androgen response elements (AREs) in the target genes’ promoter/enhancer regions. (I) Example of a typical androgen-regulated gene, the expression of which is induced when AR binds to the ARE at the promoter region. (II) *TMPRSS2:ERG* fusion—androgen binding to the ARE regulates the ERG oncogene.

The interaction between *ERG* and the androgen receptor (AR) plays an essential role in the aetiology and progression of PCa. This interaction is predominantly significant in the context of the *TMPRSS2:ERG* gene fusion, a common genetic alteration found in PCa (5). Available data suggest that *ERG* and AR collaborate to drive PCa development, with ERG modulating the AR transcriptional program and facilitating the expression of AR target genes that enhance tumour growth and survival (5). Moreover, *ERG* may indirectly influence AR signalling pathway by altering chromatin structure and accessibility, thereby promoting AR binding to DNA and transcriptional activation. In the lack of androgens, AR remains inactive in the cytoplasm, bound to the chaperone protein HSP90. Androgen binding triggers a conformational change in AR initiating it to dissociate from HSP90 and translocate into the nucleus (78). Once in the nucleus, AR binds to androgen response elements (AREs) in the promoter or enhancer regions of target genes to regulate their transcription. In normal prostate cells, this process mechanism upregulates genes, which are essential for prostate function (79). However, in PCa cells with the *TMPRSS2:ERG* fusion, androgen-bound AR aberrantly triggers the ERG oncogene leading to tumourigenesis (80). This dual role of androgen signalling pathway controlling both normal cellular functions and oncogenic signalling pathways underlines potential therapeutic targets (5). For instance, androgen deprivation therapies (ADTs), which reduce androgen levels or prevent AR role, have been reported to reduce ERG expression in *TMPRSS2:ERG* fusion-positive tumours, thus reducing their oncogenic potential (81). The association between *ERG* and AR also has significant effects for combination therapies in PCa. Combining AR-targeted therapies with agents that selectively disrupt ERG function or its downstream pathways provides potential synergistic options for treating *TMPRSS2:ERG* fusion-positive PCa (81). However, directly targeting ERG raises challenges due to its nature as a transcription factor. Despite this, ongoing research remains to explore strategies to prevent ERG–DNA binding or interrupt *ERG*–AR interactions (81). The mechanisms underlying androgen regulation of gene expression in PCa cells are illustrated in **Figure 3**.

### 3.4 ERG and PTEN crosstalk

Recent studies suggest that prostatic intraepithelial neoplasia (PIN) can advance to invasive cancer when ERG activation coincides with loss of PTEN function (82). While some studies have proposed a link between ERG expression and Gleason score, the current evidence remains inconclusive underscoring the need for further investigation. In contrast, there is stronger evidence associating reduced PTEN expression with higher Gleason scores and poor prognosis (74). Although the relationship between ERG overexpression and loss of PTEN function has not been extensively explored, studies investigating the therapeutic potential of targeting ERG and PTEN have yielded promising results (74). Furthermore, PTEN loss has been consistently associated with adverse clinical outcomes, including poor overall survival, unfavourable pathological tumour behaviour, and the development of castration-resistant and metastatic PCa (69, 83). These findings suggest that PTEN loss may represent a more clinically significant chromosomal alteration in PCa compared to *TMPRSS2:ERG* fusion emphasising its importance as a potential biomarker and therapeutic target.

### 3.5 PTEN, interleukin-6 (IL-6), and PI3K–AKT pathway interplay in PCa

Interleukin-6 (IL-6) cytokine signalling, PTEN loss, and activation of the PI3K–AKT pathway are among key contributors to the pathophysiology of PCa. Their complex relationship promotes tumour growth, boosts cell survival, and facilitates therapeutic resistance, underlining the significance of integrating molecular approaches in developing targeted therapies and improving prognostic assessment (84, 85). Chronic inflammation is closely linked to the genetic alterations and mechanisms facilitating PCa progression (86, 87). Among the key cytokine’s mediators of inflammation, IL-6 plays a key role in PCa pathogenesis by facilitating angiogenesis, tumour development, and disease progression. Pro-inflammatory cytokine, IL-6, is the most frequently associated inflammatory mediator in PCa (88). Beyond its inflammatory role, elevated IL-6 expression is strongly associated with poor clinical outcomes, treatment resistance, and advanced disease stages. The oncogenic effects of IL-6 are facilitated through IL-6 receptor (IL-6R) signalling, which promotes the PI3K–AKT pathway, thus stimulating PCa cell growth and survival rates (84, 85). This process introduces a positive feedback loop, where PTEN loss and consequent hyperactivation of the PI3K–AKT signalling pathway further upregulate IL-6 expression amplifying its oncogenic effects.

The therapeutic potential of targeting IL-6-mediated pathways is significant. Anti-IL-6 treatments, like monoclonal antibodies against IL-6 or IL-6R, may interfere with IL-6 signalling and slow the growth of PCa (84, 85). Additionally, the potential of combination therapy is highlighted by the dynamic interaction between PI3K–AKT signalling, PTEN loss, and IL-6. Strategies that target PTEN loss block the PI3K–AKT pathway and reduce IL-6 signalling together have the potential to improve PCa treatment outcomes. These combined approaches present an achievable means to improve patient outcomes and overcome treatment resistance (84, 85).

### 3.6 ERG and PTEN tumour molecular subtyping

Prostate cancer (PCa) can be classified into four distinct molecular subtypes based on IHC staining analysis of *ERG* and *PTEN* expression. These molecular subtypes include the following: (1) normal ERG with loss of *PTEN* expression (*ERG−/PTEN*−), (2) rearranged *ERG* with normal *PTEN* expression (*ERG+/PTEN*+), (3) normal *ERG* with normal *PTEN* expression (*ERG−/PTEN*+), and (4) rearranged *ERG* with loss of *PTEN* expression (*ERG+/PTEN*−) (58, 89, 90). These tumour molecular subtypes have surfaced as valuable tools for patient stratification allowing personalised approaches to treatment based on individual molecular subtypes. The prevalence of these molecular subtypes varies across different populations, as summarised in **Table 2**. Additionally, these molecular subtypes present distinct therapeutic potentials, particularly in the setting of immunotherapy and personalised treatment, providing opportunities for more precise and efficient treatment strategies (92, 93).

**Table 2:** PCa tumour molecular subtypes based on *ERG/PTEN* expression across the populations.

SN	% ERG+/PTEN+	%ERG−/PTEN−	% ERG+/PTEN−	%ERG−/PTEN+	Population	Techniques used	References
1	27.59	6.16	12.01	54.22	UK	IHC	(58)
2	–	–	68.1	–	Canada	FISH	(65)
3	–	–	28	–	Brazil	FISH	(71)
4	26	17	23	29	Brazil	FISH	(71)
5		32			Brazil	IHC	(91)
6		14	33		Brazil	IHC	(91)
7			21.8		Canada	FISH	(65)
8			46		Brazil	IHC	(91)

### 3.7 ERG−/PTEN− tumour molecular subtypes

Prostate cancer characterised by *PTEN* loss and absence of *ERG* expression (*ERG−/PTEN*−) signifies a distinct molecular subtype with profound biological and therapeutic implications (74). This subtype exhibits reduced dependence on androgen receptor (AR) signalling compared to ERG-positive tumours, contributing to resistance to AR-targeted therapies such as androgen deprivation therapy (ADT) (94). ERG−/PTEN− tumours are often more aggressive, driven by their reliance on PI3K/AKT signalling and heightened genomic instability, and are associated with poor prognosis (69, 95, 96). Notably, this subtype appears to be more prevalent in certain populations, such as African American men, who exhibit higher rates of PTEN loss and lower frequencies of *TMPRSS2:ERG* fusion highlighting potential ethnic and genetic variations in PCa subtypes (97). While ERG−/PTEN− tumours pose therapeutic challenges due to their reduced sensitivity to conventional AR-targeted therapies, they also present opportunities for novel treatment strategies (98). To address cross-pathway interactions, inhibitors that target the PI3K/AKT/mTOR pathway have shown potential, especially when combined with AR inhibitors (99). Additionally, immunotherapy, including immune checkpoint inhibitors, is a promising therapeutic approach for this subtype, as PTEN loss has been connected to immune evasion. The findings highlight how crucial it is to use both pathway-specific and immune-modulating treatment methods to treat ERG−/PTEN− tumours to enhance patient outcomes.

### 3.8 ERG+/PTEN+ tumour molecular subtypes

The *ERG+/PTEN*+ tumour molecular subtype of PCa is characterised by *ERG* rearrangements, most commonly the *TMPRSS2:ERG* fusion, and intact *PTEN* function (58). However, the occurrence of this molecular subtype varies due to the heterogeneousness of PTEN alterations, with *PTEN* loss reported in 20%–50% of cases. *ERG* rearrangements are found in nearly 40%–70% of prostate tumours (59, 60). Biologically, *ERG* rearrangements facilitate oncogenesis by promoting androgen receptor (AR)-regulated transcriptional pathways, which enhance tumour invasion and progression (100). The intact *PTEN* function in this molecular subtype helps regulate the PI3K/AKT signalling pathway differentiating it from the more aggressive *ERG+/PTEN−* molecular subtype (101, 102).

Clinically, *ERG+/PTEN*+ tumours are known to be sensitive to AR-targeted therapies, such as androgen deprivation therapy (ADT), abiraterone, and enzalutamide, which form the keystone of treatment for this tumour molecular subtype (103). Emerging therapeutic strategies, such as precision medicine approaches that target vulnerabilities in *ERG* driven pathways and DNA repair mechanisms, such as PARP inhibitors, may benefit a subset of these tumours, particularly those with additional mutations in DNA damage repair genes (104). Immune checkpoint inhibitors also represent a potential therapeutic avenue for ERG+/PTEN+ tumours, as this subtype may maintain a more immunologically active tumour microenvironment compared to *PTEN*-deficient subtypes, though the efficacy of these treatments remains under research (105, 106). Additionally, novel therapies targeting ERG-associated signalling pathways are being explored offering promising avenues for future treatment development (107). Despite these developments, several issues still need to be addressed, including the biology and clinical variation of this subtype in other populations and the need for biomarkers to direct tailored treatment. An improved understanding of the particular vulnerabilities of ERG+/PTEN+ tumours is necessary to improve treatment approaches and patient outcomes for patients with this genetic subtype of PCa (59, 60, 107).

### 3.9 ERG−/PTEN+ tumour molecular subtypes

The *ERG−/PTEN*+ tumour molecular subtype of PCa is described by the absence of *ERG* rearrangements, such as *TMPRSS2:ERG* fusions, and the retention of *PTEN* function, which is important for controlling the PI3K/AKT signalling pathway (58). This molecular subtype falls within the wide category of *ERG*-negative prostate tumours accounting for 30%–60% of PCa cases (58, 71). The occurrence of the *ERG−/PTEN*+ subtype varies throughout populations, with studies suggesting population-specific differences in disease biology. For instance, African American men demonstrate a higher frequency of *PTEN*-positive tumours and a reduced occurrence of *ERG* expression, stressing potential ethnic and genetic variations in PCa (97, 108).

The retention of *PTEN* function in *ERG−/PTEN*+ tumours distinguish them from more aggressive *PTEN*-deficient subtypes, as *PTEN* supports the regulation of cellular proliferation and survival (58, 61). Therapeutically, these molecular tumours continue to depend on androgen receptor (AR) signalling making them responsive to androgen deprivation therapy (ADT) and AR-targeted agents such as enzalutamide and abiraterone. Furthermore, the absence of *PTEN* loss may contribute to a more immunologically active tumour microenvironment fostering the possibility of exploring immunotherapies, including immune checkpoint inhibitors, potentially in combination with AR-targeted therapies (62, 103).

The lack of *ERG* expression in this molecular subtype indicates a dependency on alternative oncogenic signalling pathways emphasising the need for further research into precision medicine approaches and innovative therapeutic targets. Understanding the distinctive molecular and immunological characteristics of *ERG−/PTEN+* tumour molecular subtypes is important for developing tailored treatment strategies and improving outcomes for patients with this subtype.

### 3.10 ERG+/PTEN− tumour molecular subtypes

The presence of *ERG* rearrangements, most frequently *TMPRSS2:ERG* fusions, and the loss of *PTEN* function are characteristics of the *ERG+/PTEN*− molecular subtype of PCa (58, 109). With more invasive tumours, higher Gleason scores, and an increased likelihood to develop castration-resistant PCa, this subtype is associated with aggressive disease (109, 110). *ERG* alterations increase transcriptional activity mediated by the androgen receptor (AR), whereas *PTEN* loss interferes with the PI3K/AKT signalling pathway leading to uncontrolled cell proliferation and resistance to apoptosis (111). *ERG* rearrangements characterised by *ERG* expression, are more common in Caucasian men compared to African American and Asian men, and the occurrence of *ERG+/PTEN*− tumours varies by ethnic group. However, all ethnic groups exhibit *PTEN* loss, especially in advanced stages of the disease (60, 65). Therapeutically, AR-targeted treatments, such as abiraterone, enzalutamide, and androgen deprivation therapy (ADT), are efficient against *ERG*-driven tumours. However, PTEN loss often confers resistance by triggering alternative signalling pathways (62, 103). To address this, inhibitors targeting the PI3K/AKT/mTOR pathway are being researched, with promising results when used in combination with AR-targeted therapies (103, 112, 113). Additionally, *ERG+/PTEN*− tumours commonly exhibit immunosuppressive characteristics, such as reduced CD8+ T-cell infiltration and enhanced recruitment of myeloid-derived suppressor cells (18, 95). These characteristics reduce the efficacy of immune checkpoint inhibitors (ICIs), such as anti-PD-1/PD-L1 or anti-CTLA-4 antibodies (18), though combination therapies that simultaneously target the PI3K/AKT pathway and boost immune responses may improve outcomes in certain subtypes (18, 95).

Significant research gaps still exist despite these developments, especially in the areas of clarifying resistance mechanisms, discovering predictive biomarkers, and understanding population-specific variances. To create more individualised and successful treatment plans for patients with *ERG+/PTEN−* PCa, several issues must be resolved.

## 4 Application of *PTEN* and *TMPRSS2:ERG* gene fusion in prostate cancer

### 4.1 Prognostic and diagnostic application of TMPRSS2:ERG gene fusion

Due to the *TMPRSS2:ERG* fusion, the *ERG* gene is more expressed in PCa in both its early and late stages (5, 114, 115). It has been suggested that this fusion event is a diagnostic biomarker for PCa and is a useful tool for distinguishing tumour molecular subtypes (116). Research indicates that over 50% of PCa cases have the *TMPRSS2:ERG* fusion and consequent *ERG* overexpression (115, 117). The identification of *ERG* protein overexpression through immunostaining in PCa samples or the presence of *TMPRSS2:ERG* in prostate tissue can both be used as reliable diagnostic markers. However, the absence of *ERG* expression is not sufficient evidence that PCa is not present. *TMPRSS2:ERG* fusion may also be a potential urine-based biomarker for PCa detection, according to recent data (118, 119). The prognostic significance of the *TMPRSS2:ERG* fusion remains controversial, with studies reporting contradictory findings (28). While some research challenges its predictive utility, others suggest that it can function as a prognostic biomarker, with increased *TMPRSS2:ERG* frequency connected to worse clinical outcomes (6, 8, 120). These discrepancies may stem from variations in patient demographics, methodologies used to detect gene fusions, and the therapeutic effects on the tumour samples analysed. As a result, cautiousness is necessary when interpreting studies that emphasise the prognostic utility of the *TMPRSS2:ERG* fusion. Despite these challenges, the *TMPRSS2:ERG* fusion remains a potential therapeutic target due to its specificity to PCa and its overexpression throughout various stages of tumour progression (121). As the most common genetic alteration in PCa, targeting *TMPRSS2:ERG* at the molecular level has gained significant interest as a potential treatment strategy. Additionally, ongoing research continues to explore the *TMPRSS2:ERG* fusion gene for its potential as a different biomarker, therapeutic target, and diagnostic and prognostic indicator in PCa (121).

### 4.2 Application of PTEN as a prognostic biomarker

Evaluating the prognosis for individuals with PCa remains a key issue in disease management. Various potential molecular indicators and biomarkers are being introduced, with different institutions developing their standards for PCa risk assessment (122). Generally, blood PSA levels and Gleason scores are utilised to determine whether patients require active monitoring and treatment decisions (122). However, these markers are associated with several limitations when classifying PCa patients for adequate disease management. The Gleason grade is regarded as the most reliable predictive biopsy metric having been enhanced through modifications to the Gleason grading system (123, 124), though the amount of data obtainable through this approach is also limited, necessitating the development of cost-effective and straightforward predictive biomarkers to identify potentially aggressive prostate tumours and assist in categorising PCa patients into various predictive groups for treatment options (125, 126). According to previously published research, PTEN depletion has been linked to PCa progression through various methods such as IHC and FISH (125). PTEN loss or deficiency plays a significant role in PCa development, as evidenced by numerous studies. Several publications have also shown a direct correlation between PTEN loss and an increased risk of biochemical recurrence following prostatectomy proving useful for categorising patients with PCa into different prognosis groups for targeted treatments (68, 69, 125, 127).

## 5 Challenges and future prospects of PTEN and TMPRSS2:ERG fusion as clinical biomarkers in prostate cancer

Prostate cancer encounters significant challenges in patient classification and management selection due to its varied tumour molecular subtypes, variable tumour aggressiveness, and heterogeneous therapeutic responses (2). While tumour molecular subtyping presents a potential approach for stratifying patients and guiding personalised therapy (128), the intricacy of the disease complicates the accurate assessment of critical genetic alterations, such as *ERG* fusions and *PTEN* variants. Addressing these challenges requires the integration of multiple molecular biomarkers and comprehensive genomic profiling to better understand the underlying biology of PCa (129). Additionally, the varying susceptibility and resistance of diverse molecular subtypes to targeted therapies further complicate the clinical application of precision medicine. For example, tumours with *ERG* rearrangements or loss of *PTEN* may demonstrate different therapeutic sensitivities and resistance mechanisms necessitating tailored treatment strategies (129).

In line with this, there is great potential for enhancing risk classification, directing treatment choices, and developing tailored therapeutics for PCa by incorporating *TMPRSS2:ERG* fusion status and *PTEN* expression into clinical practice (130, 131). The goal of future research should be to clarify the molecular processes underlying *PTEN* and *ERG* dysregulation to aid in the development of tailored therapy strategies (130, 131). We anticipate that improvements in drug discovery and molecular profiling will strengthen our capacity for predicting clinical results using PTEN and *ERG* immunostaining and optimise therapeutic decisions. Precise detection of *TMPRSS2:ERG* fusions and loss of *PTEN*, particularly through reliable and cost-effective methods, such as immunohistochemistry (IHC), will be necessary for comprehensive genomic profiling (130, 131). Additionally, the development of advanced diagnostic and analytic tools to assess gene expression patterns will enable clinicians to select and monitor precise therapeutic options, eventually improving long-term patient outcomes (130, 131). These advancements underline the importance of incorporating molecular biomarkers into routine clinical practice to refine precision medicine approaches in PCa.

## 6 Conclusion

In conclusion, the application of *PTEN* and *TMPRSS2:ERG* fusion in PCa holds considerable promise for enhancing targeted treatment strategies and improving patient outcomes. The expression patterns of *PTEN* and *ERG* provide valuable insights into tumour characteristics, patient prognosis, and treatment response promising more tailored and effective therapeutic approaches. Loss-of-function affecting the phosphatase domain of *PTEN*, often associated with aggressive tumour phenotypes and poor prognosis, underscores the potential for targeted therapeutics aimed at restoring *PTEN* function or mitigating its downstream effects. Similarly, the prevalence of *ERG* overexpression in a substantial proportion of PCa presents opportunities for the development of *ERG-*targeted therapies and diagnostic tools. The interpretation and integration of tumour molecular subtyping analysis into clinical practice require further validation through robust clinical studies and the establishment of reliable, cost-effective testing methodologies. Additionally, the exploration of integrative techniques targeting multiple pathways affected by *PTEN* and *ERG* alterations holds promise for overcoming resistance mechanisms and enhancing treatment efficacy.
